# Transient ischemic attack in the practice of neurology in a low- and middle-income country

**DOI:** 10.1055/s-0045-1812036

**Published:** 2025-10-15

**Authors:** Samia Talise El Horr de Moraes, Maramelia Miranda-Alves, Leticia Rebello, Sheila O. Martins, Fabricio Oliveira Lima, Wagner Mauad Avelar, Rodrigo Bazan, Marcos C. Lange

**Affiliations:** 1 Universidade Federal do Paraná, Hospital de Clínicas, Curitiba PR, Brazil.; 2 Universidade Federal de São Paulo, Hospital de São Paulo, São Paulo SP, Brazil.; 3 Hospital de Base de Brasília, Brasília DF, Brazil.; 4 Universidade Federal do Rio Grande do Sul, Hospital de Clínicas de Porto Alegre, Porto Alegre RS, Brazil.; 5 Hospital Moinhos de Vento, Porto Alegre RS, Brazil.; 6 Hospital Geral de Fortaleza, Fortaleza CE, Brazil.; 7 Universidade Estadual de Campinas, Campinas SP, Brazil.; 8 Universidade Estadual Paulista, Faculdade de Medicina de Botucatu, Botucatu SP, Brazil.

**Keywords:** Ischemic Attack, Transient, Stroke, Surveys and Questionnaires, Neurology, Neurologic Manifestations

## Abstract

**Background:**

Transient ischemic attack (TIA) is a critical vascular event that often precedes strokes. Despite its significance, management varies widely across physicians.

**Objective:**

To evaluate the knowledge and practices of Brazilian physicians regarding TIA diagnosis and management.

**Methods:**

A survey was conducted among members of the Brazilian Academy of Neurology. It included questions about demographic information, TIA management practices, and knowledge of guidelines.

**Results:**

While most respondents were neurologists or residents, there was significant variability in hospital admission, diagnostic testing, and treatment strategies. Many physicians relied on risk stratification tools but did not consistently follow guidelines for diagnostic imaging or medication.

**Conclusion:**

These findings highlight the need for improved education and standardized protocols for TIA management in Brazil. Implementing public health policies to address these gaps could significantly reduce stroke recurrence rates and improve patient outcomes.

## INTRODUCTION

Transient ischemic attack (TIA) affects 1.19/1,000 people per year. Around 20% of these patients will have a stroke within 7 days and 30% within 30 days. Thus, this condition must be understood not only as a critical vascular event but also as the opportunity to avoid a stroke event, which may lead to future neurological disabilities. That is to say, TIA must be recognized as a stroke warning syndrome. 1

Since TIA is considered a true vascular event, like stroke, adequate investigation and management become inevitable. In fact, patients should be started on therapy, classified for cerebrovascular risk recurrence and investigated for probable mechanisms, which will allow for specialized treatment and prevention. 2 3 4

Previous studies have shown that, although most physicians admit patients with TIA for further investigation, this has not been a rule and there is a broad range of TIA evaluations. Previous surveys focused on a specific location of the world and in some small parts of the evaluation of TIA, such as the use of scores, risk stratification, as well as type of investigation or treatment. 5 6 7 Therefore, the main purpose of the present survey is to aggregate more information on how physicians in Brazil, a continental-sized country, deal with the diagnosis and management of TIA.

## METHODS

The present work was conducted in conjunction with the Brazilian Academy of Neurology (BAN), which supported the recruitment of participants and dissemination of the survey. Based on a list of members from 2021, an email survey was sent to all registered physicians. The study followed the Ethics Council definitions and presented the informed consent form duly accepted by all respondents. Anonymization was respected, preserving the personal information of individuals and hospitals when necessary.

In this study, participants were chosen through a non-probabilistic selection method. This approach made the study easier to plan and carry out because it allowed us to specifically select participants who were accessible and willing to contribute. Intentional sampling was chosen, which means directly inviting professionals who were not only easy to reach, but also relevant to this research area. This strategy streamlined the collection process and ensured that the sample was directly aligned with the research objectives.

However, the use of a non-probabilistic, convenience sample introduces risk of selection bias, as participation was limited to those with access to email and willing to respond. This may disproportionately represent individuals with a particular interest in TIA care or greater availability, limiting the generalization of these findings to neurologists in underrepresented regions, such as the North and Midwest of Brazil.

The survey was composed of 77 questions, including characterization and profile definition. In demographic and general information, we included state, city, age, sex, year of graduation, specialty, field of interest in one's specialty, if the person works in hospital that manages stroke, type of insurance accepted for the hospital, existence of exclusive beds for stroke and TIA, type of entrance of the patient in the stroke unit, and the impact of coronavirus disease 2019 (COVID-19) pandemic.

Data were coded and organized using Microsoft Excel (Microsoft Corp.), version 2302, 32 bits, and double-checked to minimize the possibility of error and to ensure data reliability. Statistical analysis was done with the free software R (R Foundation for Statistical Computing). To perform the sociodemographic characterization of the sample, descriptive analysis was performed, through the distribution of absolute frequencies and percentages, mean and standard deviations (SDs), when compatible with the type of variable (quantitative) and other measures of central tendency (median) for the qualitative variables.

## RESULTS

### Personal and hospital data

**Figure 1:**
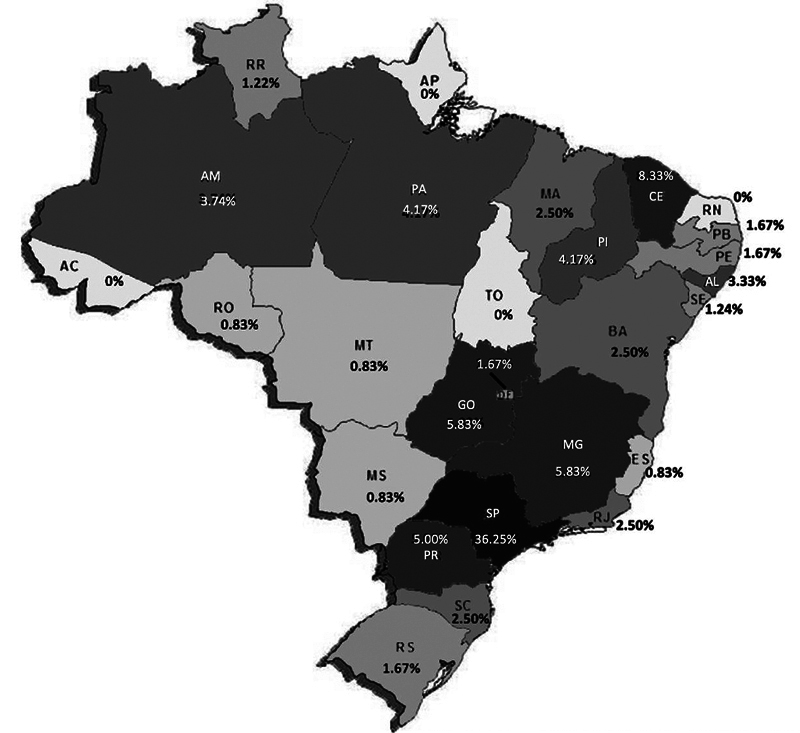
Note: The total number of people working by state exceeds the number of respondents, as some reported working in more than one state. Percentage of respondents by state in Brazil.

Only 120 professionals completed this survey, most of whom were from the Southeast region (45.4%), followed by the South region (9.17%), as presented in Figure 1 . The personal and hospital data are presented in Table 1 . The mean age was 38.7 ± 10.17 years, 78 (65%) participants were men, with 75 (62.5%) having graduated in the past 15 years. Furthermore, 108 (90%) were neurologists or residents in neurology, with 98 (82%) working in hospitals that assist patients with acute stroke and TIA. Only 38 (31%) of the hospitals had a stroke unit, and 9 (7%) had exclusive beds for TIA. Considering the COVID-19 pandemic's impact, 80 (67%) reported impact of it in the care of TIA and stroke.

**Table 1:** Physician and hospital characteristics

Characteristics		Respondents ( *n* = 120) n (%) or mean ± SD
Men		78 (65)
Age, years		38.7 ± 10.17
Time since graduation	> 15 years	35 (29.2)
	< 15 years	75 (62.5)
	Not reported	10 (8.3)
Specialty	Neurology/neurosurgery	90 (75)
	Residents in neurology or neurosurgery	18 (15)
	Emergency medicine	2 (1.7)
	Others	10 (8.3)
Work at a hospital that admits TIA/acute stroke		22 (18)
Type of health insurance	PHI	26 (18.33)
	Only SUS	47 (39.17)
	SUS and PHI	25 (20.83)
	Not reported	22 (18.33)
Exclusive beds for TIA/acute stroke		39 (33)
Stroke units		38 (32)
Exclusive beds for TIA		9 (8)

Abbreviations: PHI, private health insurance; SUS, Sistema Único de Saúde (public health care system); TIA, transient ischemic attack.

Interestingly, 53 (54%) respondents never heard about TIA clinics, while only 4 (3.3%) currently worked in one. However, 83 (69%) respondents expressed interest in having a TIA clinic to assist in their practice, 15 (12.5%) showed no interest, and 22 (18%) did not respond. Furthermore, 10 (8%) respondents treat more than 50 strokes/TIAs per month, 39 (33%) treat between 10 and 50, 31 (26%) treat between 6 and 10, and 40 (33%) treat up to 5.

### Characteristics of management of patients with TIA/acute stroke

Of all responders, 48 (40%) considered admitting all TIA patients to the hospital, while another 48 (40%) considered admitting patients based on TIA scores. Another 2 (2%) would not admit any patient, and 22 (18%) did not provide a response to this question. Considering the scores used in patient evaluation, 54 (45%) use ABCD2/3, 4 (3%) report using the National Institutes of Health (NIH) stroke scale, 5 (4%) used other scores, and 57 (47.5%) did not answer the question.

**Figure 2:**
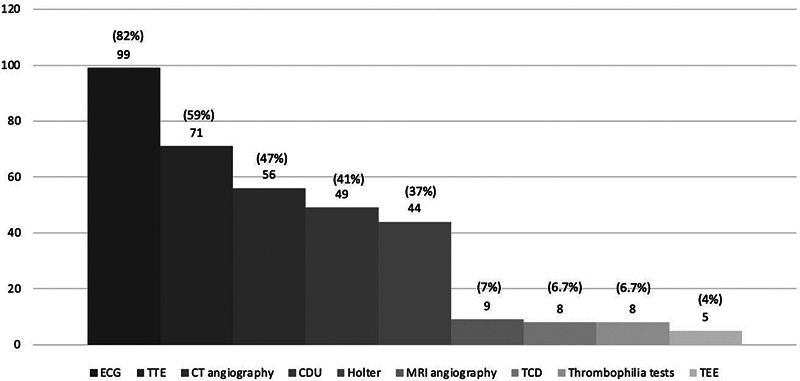
Abbreviations: ECG, electrocardiography; TTE, transthoracic echocardiography; CDU, carotid Doppler ultrasound; TCD, transcranial Doppler; TEE, transesophageal echocardiography. Complementary investigation for TIA based on the answered reported.

To investigate TIA, 92 (77%) responders stated that they request head computed tomography (CT), while 5 (4%) preferred brain magnetic resonance imaging (MRI), and 1 (1%) did not perform any brain imaging. Another 22 (18%) did not answer this question. If head CT was normal, 57 (47%) would request brain MRI. The complementary investigation is presented in Figure 2 .

In cases of non-cardioembolic TIA, 86 (72%) prescribe antiplatelet therapy for all patients, 12 (10%) refrain from initiating antiplatelet therapy for all patients, and 22 (18%) did not respond. If antiplatelet therapy is the chosen treatment, 81 (67%) would initiate treatment within 24 hours, 13 (11%) within 1 to 3 days, 1 (1%) within 14 to 30 days, and 25 (21%) did not respond. Additionally, 14 (56%) maintain treatment for up to 21 days, 9 (7%) for 22 to 90 days, 2 (2%) for 91 to 180 days, 2 (2%) for 181 to 365 days, and 67 (56%) indefinitely, with 26 (22%) not providing a response.

In terms of medication regimen, 36 (30%) prescribe monotherapy, 59 (49%) dual-antiplatelet therapy, 22 (18%) did not specify, and 3 (3%) indicated this question as not applicable. Considering only dual or triple antiplatelet therapy, 18 (15%) continue treatment for up to 21 days, 3 (2%) for 21 to 30 days, 9 (8%) for 31 to 90 days, 5 (4%) for 91 to 180 days, 5 (4%) indefinitely, and 2 (2%) indicated this question as not applicable, with 78 (65%) not providing any response.

In cases of cardioembolic TIA, 27 individuals (23%) initiate anticoagulation for all patients, 15 (12%) do not, and 78 (65%) did not provide a response. If anticoagulation is chosen for a cardioembolic TIA, 22 (18%) respondents start treatment in less than 1 day, 9 (8%) within 1 to 3 days, 4 (3%) within 4 to 6 days, 4 (3%) within 7 to 14 days, 2 (2%) without a defined timeframe, and 79 individuals (66%) did not provide this information. Likewise, respondents were asked how long they maintain anticoagulation. One (1%) mentioned maintaining it for up to 21 days, 2 (∼1%) for 91 to 180 days, 38 (32%) indefinitely, and 79 (66%) did not provide information.

Finally, of all surveyed individuals, 104 (87%) feel confident in the management of TIAs. There are 37 (31%) who do not use guidelines in their practice, while 83 (69%) followed current guidelines, including AHA/ASA for 49 (41%) of participants. When considering continuing education, 115 (96%) are interested in stroke programs/TIA.

## DISCUSSION

The present survey, conducted among members of the BAN, revealed significant gaps in the understanding and management of TIA. Although most respondents were neurologists or neurology residents, less than half hospitalized all TIA patients, indicating variability in the management approach. This is concerning, given that this condition is considered a “stroke warning,” with a high risk of progression to a full stroke within days. 8 9

Approximately 20% of TIA patients suffer a stroke within 7 days, and 30% within 30 days. 9 Therefore, it should be managed as an emergency to prevent further neurological damage. However, the reliance on risk stratification tools, such as age, blood pressure, clinical features, duration of symptoms, and diabetes (ABCD ^2^ ) or age, blood pressure, clinical features, duration, and diabetes plus dual TIA imaging (ABCD ^3^ -I), apparently leads to discrepancies in the treatment of low-risk patients. These were considered by half of the responders. This approach potentially excludes individuals who might benefit from more aggressive investigation and interventions. 8 10 11

The survey also revealed inconsistencies in the use of diagnostic imaging. Most respondents (77%) preferred head CT, even though MRI provides more detailed information, especially for detecting ischemic lesions invisible on CT. 12 Despite recognizing the importance of MRI in identifying ischemic causes, only 5% of physicians preferred it over CT. This could be due to issues of accessibility and resource availability, and it highlights a need for better dissemination of guidelines emphasizing MRI's critical role in stroke prevention. 12

One of the key methodological limitations is the high rate of missing data on crucial management decisions, particularly regarding the use of antithrombotic therapy and advanced imaging. This may affect the internal validity of the results, as the true distribution of clinical practices may be skewed. No formal sensitivity analysis or imputation method was performed to address this limitation, which should be taken into account when interpreting the findings. Future studies could benefit from strategies to minimize missing data or adopt statistical techniques to handle incomplete responses.

Around 28% of responders did not consistently prescribe antiplatelet therapy for non-cardioembolic TIA, and 77% did not prescribe anticoagulation, which is concerning given the established role of these medications in secondary stroke prevention. 13 14 15 This could significantly impact patient outcomes, as cardioembolic events are associated with a higher risk of recurrent stroke. Anticoagulation within the appropriate timeframe and under the guidance of clinical guidelines is crucial for minimizing these risks. 15

Although the study reveals substantial variability in clinical practices, it does not explore the underlying factors contributing to these differences. Future analyses could examine potential predictors of guideline adherence, such as years of clinical experience, type of healthcare institution (public vs. private), geographic region, and availability of neuroimaging or stroke units. Identifying such variables could help tailor interventions to improve clinical practice in specific contexts.

The survey results showed that most physicians start antiplatelet therapy promptly (within 24 hours), which aligns with current recommendations. 13 14 However, there is no consensus on optimal treatment length for dual antiplatelet therapy, reflecting a need for clearer guidelines and physician education.

Another notable finding was that more than half of the respondents had never heard of TIA clinics, and very few were actively working in such facilities. These clinics improve patient outcomes through rapid access to specialized care, particularly in the early stages following a TIA, when the risk of stroke is at its highest. 16 The lack of awareness of TIA clinics is an important area for improvement in the Brazilian healthcare infrastructure, and the country could benefit from the establishment of more such clinics. Interestingly, despite these gaps, most respondents expressed confidence in managing this condition. This discrepancy suggests that while physicians feel capable in their practice, there may be a mismatch between their perceived competence and adherence to best practices. This highlights the need for continued education and exposure to updated stroke and TIA management guidelines. 2 3 8 12

The findings of this survey underscore the importance of aligning clinical practices with international guidelines to ensure optimal patient care. The variability in hospital admission, diagnostic testing, and treatment strategies indicates a need for the widespread dissemination of standardized protocols for TIA management. Implementing public health policies that promote further education and training programs could significantly enhance neurologists' effectiveness.

Ensuring broader access to advanced imaging, such as MRI, and expanding the availability of TIA clinics could play a crucial role in reducing stroke recurrence rates and improving long-term outcomes. Moreover, while international guidelines such as those from the AHA/ASA are widely accepted, deviations from these protocols in Brazil may stem from resource constraints, disparities in healthcare infrastructure, and differences in national or institutional recommendations. The feasibility of uniformly implementing such protocols across Brazil is challenged by these systemic limitations, which must be considered when promoting guideline-based practices in diverse settings. As the survey revealed, while physicians show interest in continuing education, there is a need to structure these programs more effectively to bridge the existing gaps in TIA care.

The main limitations of this study include the relatively small sample size, with only 120 respondents, which may not fully represent the practices of neurologists across Brazil, particularly in less accessible or underrepresented regions, such as the North and Midwest. Additionally, the non-probabilistic sampling method, based on e-mail invitations to members of the Brazilian Academy of Neurology, may have introduced selection bias, as only physicians with greater interest or availability may have responded to the survey. The lack of responses to some critical questions, such as the use of antithrombotic therapy and diagnostic investigation, also limits the generalizability of the results. Finally, the COVID-19 pandemic may have impacted clinical practice and the participants' responses, introducing a variable that was not fully explored nor controlled for in the study.

In conclusion, this survey has shed light on critical inconsistencies in the diagnosis and management of TIA among Brazilian neurologists and neurosurgeons, as well as areas in which further education and infrastructure development could lead to improved patient outcomes. The results highlight the importance of rapid, standardized intervention following a TIA, including the use of advanced imaging and appropriate medication. A multifaceted approach is essential to support guideline implementation and improve outcomes for patients with TIA in Brazil. This could include improved physician training, enhanced infrastructure (such as broader MRI access and TIA clinics), and policy adjustments that consider regional healthcare disparities—.
